# Beyond awareness: 25 years of population data on the impact of Australia’s Beyond Blue

**DOI:** 10.1017/S2045796026100857

**Published:** 2026-07-24

**Authors:** Nicola J. Reavley, Meredith Harris, Jane Pirkis, Amy Morgan

**Affiliations:** 1 Centre for Mental Health and Community Wellbeing, Melbourne School of Population and Global Health, The University of Melbourne, Australia; 2 School of Public Health, The University of Queensland, Australia

**Keywords:** anxiety, campaigns, depression, help-seeking, mental health literacy, stigma

## Abstract

Campaigns to enhance public awareness and attitudes towards mental health problems have been a feature of mental health policy and practice for several decades. In Australia, Beyond Blue, an independent, not-for-profit organisation which was launched in 2000, marked a turning point in the national approach to improving population mental health. From the outset, it prioritised a systemic approach including national awareness campaigns, support for consumers and carers, early intervention, primary care reform and investment in research.

Over the past 25 years, Australia has seen substantial improvements in mental health literacy, stigma and help-seeking. To explore Beyond Blue’s contribution, we drew on nationally representative surveys of mental health literacy, stigma and service use. Analyses of surveys from the mid-2000s showed that respondents in states with greater exposure to Beyond Blue had higher awareness, better recognition of depression and more accurate treatment beliefs. Later studies confirmed that engagement with Beyond Blue was associated with higher mental health literacy, including among young people. National surveys have also shown significant reductions in stigma towards people with depression, changes not observed in other countries. Longitudinal data suggest that engaging with Beyond Blue predicts lower stigma and greater help-seeking over time.

National Surveys of Mental Health and Wellbeing have shown steady increases in professional help-seeking and perceived need for support since the late 1990s. While there are multiple reasons for this, recent national survey data link engagement with Beyond Blue to increased contact with health professionals and informal supports.

Globally, Beyond Blue is unique in its long-term, multi-pronged approach to improving population mental health. Its sustained focus and demonstrated impact on mental health literacy, stigma and help-seeking for depression and anxiety remain largely unmatched among comparable national initiatives.

## Introduction

Since the early 1990s, campaigns to enhance public awareness and attitudes towards mental health problems have been a feature of mental health policy and practice. This is based on the understanding that adequate recognition and treatment of mental health problems require more than better clinical care alone. Notable examples include the UK’s 1992–96 Defeat Depression Campaign (Paykel *et al.*, 1998), the European Alliance Against Depression, which was formed in 2004 and implemented in 17 European countries (Hegerl and Wittenburg, 2009) and more recently, the UK’s Time to Change anti-stigma campaign (Henderson *et al.*, 2019).

In Australia, Beyond Blue, which was launched in 2000, marked a turning point in the national approach to improving population mental health. It is an independent, not-for-profit organisation funded through a combination of Australian Government funding, state and territory contributions and philanthropic and corporate partnerships. Its staff and board include expertise from psychology, psychiatry, public health, communications, community engagement, digital services and lived experience. Its public campaigns are typically developed using social marketing and public health communication approaches, while its service and information content is informed by clinical and research evidence as well as lived experience.

Beyond Blue was formed to respond to growing calls for public engagement, stigma reduction and stronger advocacy to tackle depression. Evidence for this came from a number of areas, including Australia’s first national survey of population mental health literacy, which revealed that antidepressants and psychotherapy were regarded as less helpful for depression than vitamins and minerals and that half the population believed weakness of character was a likely cause of depression (Jorm *et al.*, 1997a, 1997b).

From the outset, Beyond Blue prioritised a systemic approach that involved national awareness campaigns, support for consumers and carers, early intervention, primary care reform and investment in strategic, applied research (Hickie, 2004). Over the last 25 years, Beyond Blue has expanded its focus beyond depression, adding anxiety conditions in 2011 and suicide prevention in 2015. In 2023, Beyond Blue sharpened its focus on earlier intervention and prevention.

Within its first 5 years, Beyond Blue had become a national leader in mental health policy advocacy, the provision of consumer and carer support and the generation and dissemination of knowledge about mental health (Pirkis *et al.*, 2005). It provides telephone and online support services, which have been shown to lead to greater engagement with health professionals, reduced feelings of distress, increased confidence to cope and less hopelessness (Hawkins *et al.*, 2020). As an incubator, it has been central to the development, trialling and dissemination of early intervention programmes. Initial investments in school-based programmes (Spence *et al.*, 2005; Sawyer *et al.*, 2010) have since evolved into the Australian National Mental Health in Education Initiative, *Be You*, which is freely available to all early learning services, primary and secondary schools throughout Australia (Hoare *et al.*, 2020). Beyond Blue also played a key role in changing approaches to workplace mental health in Australia, including through their National Mental Health and Wellbeing Study of Police and Emergency Services (Kyron *et al.*, 2021). More recent initiatives include The Way Back suicide aftercare service, which led to improved wellbeing and reduced suicidality and psychological distress in service users (Nous Group, 2022), and NewAccess low-intensity therapies for symptoms of depression and anxiety, which led to clinically meaningful reductions in depression and anxiety symptoms (Baigent *et al.*, 2023).

From its earliest days, Beyond Blue has been guided by Blue Voices, a lived and living experience reference group and online community. Now numbering over 3,000 individuals, membership is open to Australians aged 16 or older who have personally experienced a mental health condition, supported someone with one or been affected by suicide. Through surveys, focus groups, advisory and governance committees, co-design of products and services and consultation on policy and campaign design, Blue Voices has played a central role in facilitating consumer and carer participation in planning, delivering and evaluating mental health services and activities to improve mental health literacy and reduce stigma and discrimination (Pirkis *et al.*, 2005).

Globally, among national government-funded initiatives to improve mental health literacy, reduce stigma and discrimination and facilitate help-seeking for depression and anxiety, Beyond Blue stands out for its sustained efforts and evidence of impact. As we reflect on its 25-year milestone, we consider what the landscape might look like had Beyond Blue not existed. To do this, we can draw on Beyond Blue’s five independent external evaluations, which were consistently positive (Pirkis *et al.*, 2005; Dunt *et al.*, 2010; Siggins Miller Consultants, 2018; ARTD Consultants, 2023; Blue, 2023) as well as a range of nationally representative surveys on mental health and wellbeing, service use, mental health literacy and stigma.

## Awareness of Beyond Blue

From its earliest days, Beyond Blue actively promoted media coverage of depression and directly contributed to an increase in the quality and quantity of information available through media and educational sources (Pirkis *et al.*, 2005). Community awareness of Beyond Blue was assessed in national telephone surveys from as early as 2001, with 22% of Australians recognising the brand name in that year. No discussion of the early history of Beyond Blue would be complete without mention of the Hon. Jeff Kennett AC, the former premier of Victoria, who founded Beyond Blue and played a central role in securing funds for a mental health advocacy group focusing on depression. The 2003/4 National Survey of Mental Health Literacy and Stigma (Jorm *et al.*, 2005a) included a question asking whether respondents were aware of Beyond Blue, with 24% of people saying yes and a further 9% saying yes when prompted with information about his involvement. While its activities were principally aimed at adults, awareness of Beyond Blue among young people was also relatively high. In a 2006 national survey of young people aged 12–25 years, 44% were aware of Beyond Blue (Morgan and Jorm, 2007).

By 2011, recognition of Beyond Blue was 78% (Reavley and Jorm, 2011). More recent surveys put this at 94%, and it remains the most recognised mental health organisation in Australia (Beyond Blue and The Social Research Centre, 2025). In 2024, up to one in six Australians had connected with Beyond Blue’s content and services over the previous 12 months. This included visiting the website, reading a brochure, following on social media or engaging with Beyond Blue support services on the phone, via webchat or forums, or the NewAccess programme.

## Beyond Blue and improvements in mental health literacy, stigma reduction and increases in access to care

However, high awareness of a mental health organisation counts for little unless it leads to tangible outcomes such as greater mental health literacy, reduced stigma and meaningful engagement with care. Evidence points to the impact of Beyond Blue in these key areas.

### Mental health literacy

The last 25 years have seen considerable improvements in mental health literacy in the Australian population (Reavley and Jorm, 2012a; Nicholas *et al.*, 2019; Reavley *et al.*, 2025). While several efforts are likely to have contributed to these improvements, early evidence of Beyond Blue’s role came from data from the 1995 and 2003/4 National Surveys of Mental Health Literacy and Stigma. Analyses took advantage of the fact that Beyond Blue was rolled out in some states (principally Victoria) and not others (notably NSW and WA), allowing for comparison between high-exposure and low-exposure states. In the former, awareness of Beyond Blue was found to be around twice the level of the latter (Jorm *et al.*, 2005b). Recognition of depression and beliefs about treatments improved greatly at a national level, but slightly more so in the high-exposure states (Jorm *et al.*, 2006).

More recent nationally representative studies also point to the associations between awareness of Beyond Blue and higher levels of mental health literacy, including in young people. Surveys in 2006 and 2011 showed associations between recognition of Beyond Blue and more accurate recognition of mental health problems, more favourable beliefs about evidence-based treatments and self-help intervention and beliefs about the helpfulness of first-aid actions that were more closely aligned with professional ratings (Morgan and Jorm, 2007; Yap *et al.,* 2012a, 2012b, 2012c).

### Stigma and discrimination

Driven by the priorities of consumers, as well as early research findings showing that many people believed in personal weakness as a cause of depression (Jorm *et al.*, 1997b), Beyond Blue has had a focus on stigma reduction since its earliest days. Beyond Blue’s speakers programme plays a central role in its stigma reduction activities, with over 500 speaking and media events per year reaching over 6 million people Australia-wide. As with mental health literacy, there have been changes over the last 25 years. The 2003/4 and 2011 National Surveys of Mental Health Literacy and Stigma included questions about attitudes and willingness to interact with a person with depression. Analysis of changes over that time period showed decreases in the proportions of people believing that a person with depression was personally weak or could ‘snap out of’ the problem, while there were minimal changes in attitudes to a person with schizophrenia (Reavley and Jorm, 2012).

Analysis of data from a more recent nationally representative survey shows further changes since 2011, most notably in relation to large reductions in beliefs about the dangerousness of a person with depression (Reavley *et al.*, 2026). These changes have not been seen in other countries (Pescosolido *et al.*, 2021; Schomerus *et al.*, 2022). Recognition of Beyond Blue was associated with less stigmatising beliefs in this survey and similar findings have previously been seen in young people (Yap *et al.*, 2013). Further support for the anti-stigma role of Beyond Blue comes from its Australia’s Mental Health and Wellbeing Check Surveys, which were conducted in 2022 and 2024 (unpublished data). Because this survey recruited participants from The Social Research Centre’s Life in Australia panel, 2,500 people took part at both time points, enabling us to conduct longitudinal analysis. Engagement with Beyond Blue’s content in 2022 (such as visiting the website, reading a brochure or following on social media) predicted significantly lower stigma related to seeking professional help 2 years later (controlling for stigma, mental health symptoms and self-reported mental health problems in 2022). While these attitudinal changes are positive, a key measure of success relates to how they translate into improvements in access to care or experiences of discrimination or support. While relatively few studies measure these, evidence from nationally representative studies of experiences of discrimination and support in Australia points to a lack of progress in some settings, such as workplaces (Reavley *et al.*, 2026). New approaches are needed, with interventions carefully developed and disseminated based on their ability to bring about tangible behaviour change in their target audiences.

### Help-seeking for mental health conditions

A core aim of Beyond Blue since its inception has been to promote help-seeking for mental health conditions, particularly by improving mental health literacy and decreasing stigma related to earlier engagement with support services.

Comparisons based on published data from Australia’s 2020–22 National Study of Mental Health and Wellbeing (NSMHWB) and its precursors in 1997 and 2007 indicate shifts in help-seeking for mental health among Australian adults since the inception of Beyond Blue. In the 1997 NSMHWB, 11% of people said they had consulted a health professional for their mental health in the past 12 months (Meadows *et al.*, 2001); in the 2007 and 2020–22 surveys, these proportions were 12% and 17%, respectively (Harris *et al.*, 2025). In addition, published data suggest that the proportion of Australian adults who perceived a need for information about mental illness, treatment and services increased from 5% in 1997 to 8% in 2007 and 11% in 2020–22; perceived need for counselling increased from 10% in 1997 to 11% in 2007 and 15% in 2020–22 (Meadows *et al.*, 2000; Meadows and Burgess, 2009; Australian Bureau of Statistics, 2023).

In the context of these broader population shifts, other sources of evidence indicate that Beyond Blue has influenced attitudes to help-seeking as well as actual rates of help-seeking. As discussed above, national mental health literacy survey data allowed for comparisons between high Beyond Blue-exposure and low-exposure states. Respondents in high-exposure states showed greater positive change in beliefs about the benefits of help-seeking in general and those related to counselling and medication (Jorm *et al.*, 2005a). More recent data also support the impact on help-seeking attitudes. Beyond Blue’s Mental Health and Wellbeing Check Surveys in 2022 and 2024 showed that engaging with Beyond Blue content or services in 2022 significantly improved attitudes to seeking help for a personal or emotional problem from a mental health professional or doctor 2 years later, controlling for attitudes in 2022. Moreover, in 2024, 27% of participants reported that, as a result of their engagement with Beyond Blue services (e.g. phone support service), they had spoken with their doctor/GP about mental health, 21% reported they had sought professional support from a psychologist or other health professional and 33% reported they had spoken to family or friends about mental health. Analysis of the matched longitudinal sample from the 2022 and 2024 Mental Health and Wellbeing Check Surveys also supports Beyond Blue’s impact on actual help-seeking. Engagement with Beyond Blue in 2022 significantly increased rates of seeking professional help 2 years later, controlling for help-seeking in 2022. Help-seeking rates were 28% for those who engaged with Beyond Blue versus 20% who reported no engagement.

## Globally unique scale and impact

Beyond Blue is globally unique among national initiatives that have aimed to use a systemic and multi-pronged approach to improve population mental health. Beyond Blue’s sustained focus and measurable success in improving mental health literacy, reducing stigma and facilitating help-seeking for depression and anxiety remains largely unmatched. While other countries, including the UK and Canada, have had national anti-stigma or awareness campaigns, these often lack rigorous evaluation of population-level effects or have shown limited impacts on mental health literacy or mental health and wellbeing (Hahn *et al.*, 2023). An exception might be the European Alliance Against Depression, a multi-component intervention with activities targeted to primary care, the general public, community facilitators and affected individuals and their families. This has been shown to be effective in reducing mental health-related outcomes such as suicidal behaviours in several European countries (Arensman *et al.*, 2026).

In the area of stigma reduction, the UK’s Time to Change anti-stigma campaign, which comprised social marketing, lived experience contact, community and workplace programmes, and which ran from 2007 to 2021, may be the closest example to Beyond Blue. This was evaluated in national surveys, which showed some evidence of reduced stigma and discrimination, but also that sustained effects have been challenging to maintain after the end of the programme (Henderson *et al.*, 2019; Ronaldson and Henderson, 2024). These findings, along with those showing limited changes in other countries, point to the importance of strategic, multi-pronged and sustained efforts at a national scale of the type that Beyond Blue exemplifies.

## Where to next?

As Beyond Blue looks to its next 5 years, it has resolved to sharpen its focus on prevention and early intervention (Beyond Blue, 2023). This reflects the growing evidence that, despite the increased rates of service use seen in the recent NSMHWB, there have been limited reductions in the prevalence rates of depression and anxiety and, in young adults and adolescents, these may even be increasing (Slade *et al.*, 2025). These findings have led to calls to balance a health sector response with a greater focus on prevention, early intervention and mental health promotion (Productivity Commission June 2025). Beyond Blue is well placed to build on its previous work on addressing key social determinants of mental health through campaigns (e.g., the Invisible Discriminator campaign (Beyond Blue, 2019)); partnerships with organisations such as Financial Counselling Australia, the Australian Securities and Investments Commission and Adult Migrant Employment Services; and advocacy on issues that present key risks to mental health such as financial distress, racial discrimination, loneliness and the design of social media applications.

Key challenges for mental health systems are also likely to arise from broader societal issues, including the lingering effects of the Covid pandemic, cost-of-living-related challenges, the mental health impacts of climate change and the risks and opportunities posed by artificial intelligence. Improving population mental health may also require engagement with ongoing debates about the expanding boundaries of mental illness and the medicalisation of everyday distress, which may carry unintended risks, including pathologising common emotional responses, encouraging overreliance on clinical services and possibly increasing psychological distress (Foulkes and Andrews, 2023; Reavley *et al.*, 2025). It is possible that the simplistic messaging included in many public-facing campaigns may have inadvertently contributed to these dynamics, pointing to the importance of designing interventions that pair help-seeking messages with non-clinical support options. There is also some evidence that a focus on depression and anxiety conditions has widened the gaps in mental health literacy and stigma towards people with other conditions, potentially worsening inequalities between people with less severe and more severe conditions (Morgan *et al.*, 2026; Reavley, 2026). In recognition of these issues, in recent years, Beyond Blue has moved away from labelling of discrete conditions and is more focused on supporting people to recognise signs of emerging distress and reassuring them that support is available.

At the same time, as a society, we must take care not to further entrench gaps in care for those who meet diagnostic criteria for mental health conditions but remain disconnected from help. Beyond Blue’s strategic, large-scale impactful approaches to improving population mental health position it well to navigate these challenging emerging issues.

## Data Availability

Data may be made available on request.

## References

[ref1] Arensman E, Sadath A, Callanan A, Khan A, Leduc M, Cully G, McTernan N, Schnitzspahn K, Abdulla K, Celik S, Hauck P, Pina C, Giupponi G, Roberti M, Conca A, Nuhara V, Lazzeri M, Trentin S, Tosti M, Belfanti A, Ferrara C, Sola VP, Allende S, Diaz AJ, Székely A, Ruzsa D, Zsák É, Székely A, Jr, Toczyski P, Van Audenhove C, Coppens E, Fexi G, Deredini P, Konsta N, Arabatzis T, Pasho B, Tsagaraki E, Naydenova T, Drobachka A, Värnik P, Sirg A, Sisask M, Lillepuu L, Mere R and Hegerl U (2026) The European Alliance Against Depression approach: An evidence-based program to reduce depression and suicidal behavior. *Nature Mental Health* 4(1), 42–51.

[ref2] ARTD Consultants (2023) Beyond Blue: Independent Evaluation. https://edge.sitecorecloud.io/beyondblue1-beyondblueltd-p69c-fe1e/media/Project/Sites/beyondblue/PDF/About/Beyond-Blue-Independent-Evaluation–FINAL-Full-Report-2023.pdf (accessed 6 November 2025).

[ref3] Australian Bureau of Statistics (2023) National Study of Mental Health and Wellbeing: Tables 1, 12 and 13. https://www.abs.gov.au/statistics/health/mental-health/national-study-mental-health-and-wellbeing/latest-release#data-downloads (accessed 20 October 2025).

[ref4] Baigent M, Smith D, Battersby M, Lawn S, Redpath P and McCoy A (2023) The Australian version of IAPT: Clinical outcomes of the multi-site cohort study of NewAccess. *Journal of Mental Health* 32(1), 341–350. 32394756 10.1080/09638237.2020.1760224

[ref5] Beyond Blue (2023) Strategy 2023+. Earlier. Easier. Together. https://www.beyondblue.org.au/about/strategy (accessed 16 October 2025).

[ref6] Beyond Blue and The Social Research Centre (2025) Australia’s Mental Health and Wellbeing Check: Trends in mental health and support-seeking. https://edge.sitecorecloud.io/beyondblue1-beyondblueltd-p69c-fe1e/media/Aust_MH_and_Wellbeing_Check_02JUN25.pdf (accessed 19 October 2025).

[ref7] Blue B. (2019) The Invisible Discriminator. https://www.youtube.com/watch?v=7FUdrd0Mg_4 (accessed 6 November 2025).

[ref8] Blue B. (2023) Beyond Blue: Independent Evaluations. https://www.beyondblue.org.au/about/research-projects (accessed 16 October 2025).

[ref9] Dunt D, Robinson J and Pirkis J (2010) *Evaluation of Beyond Blue*. Melbourne: The University of Melbourne.

[ref10] Foulkes L and Andrews JL (2023) Are mental health awareness efforts contributing to the rise in reported mental health problems? A call to test the prevalence inflation hypothesis. *New Ideas in Psychology* 69, 101010.

[ref11] Hahn JS, Chua K-C, Jones R and Henderson C (2023) The Every Mind Matters campaign: Changes in mental health literacy and its associations with campaign awareness. *European Journal of Public Health* 33(6), 1008–1013. 37579223 10.1093/eurpub/ckad145 PMC10710357

[ref12] Harris MG, Tapp C, Vescovi JJ, Sunderland M, Diminic S, Chapman C, Slade TN, Teesson M, Pirkis J and Burgess PM (2025) Consultation with health professionals for mental health in Australia in 2020–2022 and changes since 2007: Findings from the 2020–2022 National Study of Mental Health and Wellbeing. *Australian & New Zealand Journal of Psychiatry* 59(9), 810–823. 40660899 10.1177/00048674241307919 PMC12397540

[ref13] Hawkins A, Odgers J, Reeves A and McCoy A (2020) Evaluating telephone and online psychological support and referral. *Evaluation Journal of Australasia* 20(3), 157–175.

[ref14] Hegerl U and Wittenburg L (2009) Focus on mental health care reforms in Europe: The European alliance against depression: A multilevel approach to the prevention of suicidal behavior. *Psychiatric Services* 60(5), 596–599. 19411345 10.1176/ps.2009.60.5.596

[ref15] Henderson C, Potts L and Robinson EJ (2019) Mental illness stigma after a decade of Time to Change England: Inequalities as targets for further improvement. *European Journal of Public Health* 30(3), 497–503. 10.1093/eurpub/ckaa013 PMC7292343 32531039

[ref16] Hickie I (2004) Can we reduce the burden of depression? The Australian experience with beyondblue: The national depression initiative. *Australas Psychiatry* 12(1_suppl), S38–46. 15715829 10.1080/j.1039-8562.2004.02097.x-2

[ref17] Hoare E, Thorp A, Bartholomeusz-Raymond N, McCoy A, Butler H and Berk M (2020) Be You: A national education initiative to support the mental health of Australian children and young people. *Australian and New Zealand Journal of Psychiatry* 54(11), 1061–1066. 32794411 10.1177/0004867420946840

[ref18] Jorm AF, Christensen H and Griffiths KM (2006) Changes in depression awareness and attitudes in Australia: The impact of beyondblue: The national depression initiative. *Australian & New Zealand Journal of Psychiatry* 40(1), 42–46. 16403036 10.1080/j.1440-1614.2006.01739.x

[ref19] Jorm AF, Korten AE, Jacomb PA, Christensen H, Rodgers B and Pollitt P (1997a) “Mental health literacy”: A survey of the public’s ability to recognise mental disorders and their beliefs about the effectiveness of treatment. *Medical Journal of Australia* 166(4), 182–186. 9066546 10.5694/j.1326-5377.1997.tb140071.x

[ref20] Jorm AF, Korten AE, Jacomb PA, Christensen H, Rodgers B and Pollitt P (1997b) Public beliefs about causes and risk factors for depression and schizophrenia. *Social Psychiatry and Psychiatric Epidemiology* 32(3), 143–148. 9130866 10.1007/BF00794613

[ref21] Jorm AF, Griffiths KM and Griffiths KM (2005a) The impact of beyondblue: The national depression initiative on the Australian Public’s recognition of depression and beliefs about treatments. *Australian & New Zealand Journal of Psychiatry* 39(4), 248–254. 15777361 10.1080/j.1440-1614.2005.01561.x

[ref22] Jorm AF, Nakane Y, Christensen H, Yoshioka K, Griffiths KM and Wata Y (2005b) Public beliefs about treatment and outcome of mental disorders: A comparison of Australia and Japan. *BMC Medicine* 3(1), 12. 16004615 10.1186/1741-7015-3-12 PMC1177951

[ref23] Kyron MJ, Rikkers W, Page AC, O’Brien P, Bartlett J, LaMontagne A and Lawrence D (2021) Prevalence and predictors of suicidal thoughts and behaviours among Australian police and emergency services employees. *Australian & New Zealand Journal of Psychiatry* 55(2), 180–195. 32615800 10.1177/0004867420937774

[ref24] Meadows G, Burgess P, Fossey E and Harvey C (2000) Perceived need for mental health care, findings from the Australian National Survey of Mental Health and Well-being. *Psychological Medicine* 30(3), 645–656. 10883719 10.1017/s003329179900207x

[ref25] Meadows G, Liaw T, Burgess P, Bobevski I and Fossey E (2001) Australian general practice and the meeting of needs for mental health care. *Social Psychiatry and Psychiatric Epidemiology* 36(12), 595–603. 11838831 10.1007/s127-001-8199-7

[ref26] Meadows GN and Burgess PM (2009) Perceived need for mental health care: Findings from the 2007 Australian survey of mental health and wellbeing. *Australian & New Zealand Journal of Psychiatry* 43(7), 624–634. 19530019 10.1080/00048670902970866

[ref27] Morgan AJ and Jorm AF (2007) Awareness of beyondblue: The national depression initiative in Australian young people. *Australasian Psychiatry* 15(4), 329–333. 17612888 10.1080/10398560701323976

[ref28] Morgan AJ, Ross AM, McNaught G, Green R and Reavley NJ (2026) Public stigma of mental illness in Australia: Shifts in attitudes across nationally representative surveys in 2011 and 2024. *Epidemiology and Psychiatric Sciences* 35, e26. 41983280 10.1017/S2045796026100602 PMC13122537

[ref29] Nicholas A, Pirkis J, Jorm A, Spittal MJ and Reavley N (2019) Helping actions given and received in response to suicide risk: Findings from an Australian nationally representative telephone survey. *SSM - Population Health* 9, 100483. 31646168 10.1016/j.ssmph.2019.100483 PMC6804460

[ref30] Nous Group (2022) The Way Back Support Services Evaluation: Final Evaluation Report. Sydney, Australia: Nous Group.

[ref31] Paykel ES, Hart D and Priest RG (1998) Changes in public attitudes to depression during the Defeat Depression Campaign. *British Journal of Psychiatry* 173(6), 519–522. 10.1192/bjp.173.6.519 9926082

[ref32] Pescosolido BA, Halpern-Manners A, Luo L and Perry B (2021) Trends in public stigma of mental illness in the US, 1996-2018. *JAMA Network Open* 4(12), e2140202. 34932103 10.1001/jamanetworkopen.2021.40202 PMC8693212

[ref33] Pirkis J, Hickie I, Young L, Burns J, Highet N and Davenport T (2005) An evaluation of beyondblue, Australia’s national depression initiative. *International Journal of Mental Health Promotion* 7(2), 35–53.

[ref34] Productivity Commission (June 2025) *Mental Health and Suicide Prevention Agreement Review, Interim Report*. Canberra: Productivity Commission.

[ref35] Reavley NJ (2026) Rethinking stigma reduction programs for people with severe mental illness. *World Psychiatry* 25(1), 86–87. 41536094 10.1002/wps.70007 PMC12805037

[ref36] Reavley NJ and Jorm AF (2011) Recognition of mental disorders and beliefs about treatment and outcome: Findings from an Australian national survey of mental health literacy and stigma. *Australian & New Zealand Journal of Psychiatry* 45(11), 947–956. 21995330 10.3109/00048674.2011.621060

[ref37] Reavley NJ and Jorm AF (2012a) Public recognition of mental disorders and beliefs about treatment: Changes in Australia over 16 years. *British Journal of Psychiatry* 200(5), 419–425. 10.1192/bjp.bp.111.104208 22442098

[ref38] Reavley NJ and Jorm AF (2012b) Stigmatizing attitudes towards people with mental disorders: Changes in Australia over 8 years. *Psychiatry Research* 197(3), 302–306. 22417929 10.1016/j.psychres.2012.01.011

[ref39] Reavley NJ, Jorm AF, Carbone S, Tsiamis E and Morgan AJ (2025) Testing the diagnostic expansion hypothesis with a population-based survey of attitudes to depression in Australia. *BMJ Public Health* 3(2), e003040. 40937426 10.1136/bmjph-2025-003040 PMC12421594

[ref40] Reavley NJ, Ross AM, Green R, McNaught G and Morgan AJ (2026) Nationally representative surveys of experiences of discrimination and positive treatment in people with mental health problems in Australia: Changes over 10 years. *Social Science & Medicine* 400, 119215. 42000620 10.1016/j.socscimed.2026.119215

[ref41] Ronaldson A and Henderson C (2024) Investigating changes in mental illness stigma and discrimination after the Time to Change programme in England. *BJPsych Open* 10(6), e199. 39501845 10.1192/bjo.2024.801 PMC11698152

[ref42] Sawyer MG, Pfeiffer S, Spence SH, Bond L, Graetz B, Kay D, Patton G and Sheffield J (2010) School-based prevention of depression: A randomised controlled study of the beyondblue schools research initiative. *Journal of Child psychology and Psychiatry* 51(2), 199–209. 19702662 10.1111/j.1469-7610.2009.02136.x

[ref43] Schomerus G, Schindler S, Sander C, Baumann E and Angermeyer MC (2022) Changes in mental illness stigma over 30 years - Improvement, persistence, or deterioration? *European Psychiatry* 65(1), e78. 36328960 10.1192/j.eurpsy.2022.2337 PMC9724218

[ref44] Siggins Miller Consultants (2018) *Beyond Blue Evaluation 2015–2018*. Sydney, Australia: Siggins Miller Consultants.

[ref45] Slade T, Vescovi J, Chapman C, Teesson M, Arya V, Pirkis J, Harris MG, Burgess PM, Santomauro D, O’Dean S, Tapp C and Sunderland M (2025) The epidemiology of mental and substance use disorders in Australia 2020-22: Prevalence, socio-demographic correlates, severity, impairment and changes over time. *Australian & New Zealand Journal of Psychiatry* 59(6), 510–521. 39245901 10.1177/00048674241275892 PMC12102513

[ref46] Spence S, Burns J, Boucher S, Glover S, Graetz B, Kay D, Patton G and Sawyer M (2005) The beyondblue schools research initiative: Conceptual framework and intervention. *Australas Psychiatry* 13(2), 159–164. 15948913 10.1080/j.1440-1665.2005.02180.x

[ref47] Yap MB, Reavley N and Jorm AF (2012a) Young people’s beliefs about preventive strategies for mental disorders: Findings from two Australian national surveys of youth. *Journal of Affective Disorders* 136(3), 940–947. 21975138 10.1016/j.jad.2011.09.003

[ref48] Yap MB, Reavley N, Mackinnon AJ and Jorm AF (2013) Psychiatric labels and other influences on young people’s stigmatizing attitudes: Findings from an Australian national survey. *Journal of Affective Disorders* 148(2-3), 299–309. 23333077 10.1016/j.jad.2012.12.015

[ref49] Yap MB, Reavley NJ and Jorm AF (2012b) Associations between awareness of beyondblue and mental health literacy in Australian youth: Results from a national survey. *Australian & New Zealand Journal of Psychiatry* 46(6), 541–552. 22679206 10.1177/0004867411435288

[ref50] Yap MBH, Reavley NJ and Jorm AF (2012c) Intentions and helpfulness beliefs about first aid responses for young people with mental disorders: Findings from two Australian national surveys of youth. *Journal of Affective Disorders* 136(3), 430–442. 22137764 10.1016/j.jad.2011.11.006

